# Supplementary material for: Diversity of compounds in femoral secretions of Galápagos iguanas (genera: Amblyrhynchus and Conolophus), and their potential role in sexual communication in lek-mating marine iguanas (Amblyrhynchus cristatus)
Source: PeerJ. 2017 Aug 17;5:e3689. doi: 10.7717/peerj.3689 (PMC5563446; doi:10.7717/peerj.3689)
Supplement: Supplemental Information 2 [file peerj-05-3689-s002.docx]

**Table S1** Sampled populations of *A. cristatus* and their coordinates. “La Lobería” (SRL) and “Punta Pitt” (SRPC) are considered different populations in recognition of their genetic divergence (MacLeod et al. 2015).

| **Island** | **Coordinates** | **Abbreviation** |
| --- | --- | --- |
| Genovesa | N 0.31065, W 89.97349 | GEN |
| Marchena | N 00.30051, W 090.50774 | MAR |
| Pinta | N 00. 54340, W 090.73948 | PIN |
| Santiago | S 00.24215, W 090.86495 | SAN |
| Santa Cruz | S 00. 74180, W 090. 30732 | CRUZ |
| San Cristóbal (La Lobería) | S 00.92214, W 089.62125 | SRL |
| San Cristóbal (Punta Pitt) | S 00.71432, W 089.24174 | SRPC |
| Española | S 01.39502, W 089.62029 | ESP |
| Floreana | S 01.31968, W 090.50911 | FL |
| Santa Fe | S 00.82581, W 090.02861 | SFE |
| Fernandina | S 00.44264 W 091.38947 | FDA |
| Isabela | S 00.78524 W 091.42460 | IS |
