# Supplementary material for: Diversity of compounds in femoral secretions of Galápagos iguanas (genera: Amblyrhynchus and Conolophus), and their potential role in sexual communication in lek-mating marine iguanas (Amblyrhynchus cristatus)
Source: PeerJ. 2017 Aug 17;5:e3689. doi: 10.7717/peerj.3689 (PMC5563446; doi:10.7717/peerj.3689)
Supplement: Supplemental Information 3 [file peerj-05-3689-s003.docx]

**Table S2**

Results of the principal component analysis (PCA), showing the factor loadings of each principal component (PC) for the lipophilic compounds. PCAs were run prior to the development of the models to explore lipid functionality in intraspecific communication of marine iguanas, in order to investigate the multicollinearity among independent variables. Values in bold show the higher values of coefficient loadings that indicate those chemical compounds that were selected as independent variables for subsequent model development.

|  | PC1 | PC2 | PC3 | PC4 |
| --- | --- | --- | --- | --- |
| Hexadecanoic acid | 0.23 | **0.90** | 0.11 | 0.04 |
| 9-Octadecenoic acid | 0.25 | 0.31 | 0.12 | **-0.79** |
| 11-Octadecenoic acid | 0.27 | -0.01 | 0.50 | 0.53 |
| Octadecanoic acid | 0.23 | 0.80 | -0.31 | 0.14 |
| 5,8,11,14-Eicosatetraenoic acid | 0.25 | 0.47 | -0.21 | -0.37 |
| 11-Eicosenoic acid | 0.07 | -0.04 | **0.89** | -0.05 |
| 13-Eicosenoic acid | 0.04 | 0.17 | 0.88 | 0.05 |
| Eicosanoic acid | 0.23 | 0.39 | 0.06 | 0.76 |
| Docosanoic acid | -0.87 | -0.04 | -0.07 | 0.02 |
| Tetracosanoic acid | **-0.89** | -0.01 | -0.01 | 0.02 |
| Cholesterol | 0.17 | -0.88 | -0.28 | 0.15 |
| Cholestanol | -0.78 | -0.29 | 0.08 | -0.14 |
| Cholestanone | -0.73 | -0.14 | -0.33 | 0.04 |
