# Supplementary material for: Diversity of compounds in femoral secretions of Galápagos iguanas (genera: Amblyrhynchus and Conolophus), and their potential role in sexual communication in lek-mating marine iguanas (Amblyrhynchus cristatus)
Source: PeerJ. 2017 Aug 17;5:e3689. doi: 10.7717/peerj.3689 (PMC5563446; doi:10.7717/peerj.3689)
Supplement: Supplemental Information 4 [file peerj-05-3689-s004.docx]

**Table S3**

PERMANOVA pairwise comparisons of lipid profiles across populations (n=103). Adjusted P values are shown (significant differences are highlighted in bold).

|  | IS | MAR | PIN | GEN | SAN | ESP | FL | SFE | FDA | CRUZ | SRL | SRPC |
| --- | --- | --- | --- | --- | --- | --- | --- | --- | --- | --- | --- | --- |
| IS | - | **0.005** | **0.005** | **0.003** | **0.003** | **0.003** | **0.025** | **0.005** | **0.005** | 0.110 | 0.053 | 0.053 |
| MAR | **0.005** | - | 0.544 | 0.619 | 0.771 | 0.200 | **0.003** | 0.306 | 0.053 | **0.005** | **0.005** | 0.477 |
| PIN | **0.005** | 0.544 | - | 0.477 | 0.132 | 0.332 | **0.006** | **0.032** | **0.003** | **0.003** | **0.005** | 0.116 |
| GEN | **0.003** | 0.619 | 0.477 | - | 0.058 | 0.069 | **0.003** | 0.151 | **0.003** | **0.003** | **0.003** | 0.306 |
| SAN | **0.003** | 0.771 | 0.132 | 0.058 | - | 0.065 | **0.003** | **0.008** | **0.005** | **0.003** | **0.003** | 0.176 |
| ESP | **0.003** | 0.200 | 0.332 | 0.069 | 0.065 | - | **0.003** | **0.005** | **0.003** | **0.003** | **0.003** | **0.022** |
| FL | **0.025** | **0.003** | **0.006** | **0.003** | **0.003** | **0.003** | - | **0.003** | **0.003** | **0.006** | **0.010** | **0.006** |
| SFE | **0.005** | 0.306 | **0.032** | 0.151 | **0.008** | **0.005** | **0.003** | - | 0.085 | **0.013** | **0.005** | 0.784 |
| FDA | **0.005** | 0.053 | **0.003** | **0.003** | **0.005** | **0.003** | **0.003** | 0.085 | - | **0.031** | 0.098 | 0.493 |
| CRUZ | 0.110 | **0.005** | **0.003** | **0.003** | **0.003** | **0.003** | **0.006** | **0.013** | **0.031** | - | 0.337 | 0.314 |
| SRL | 0.053 | **0.005** | **0.005** | **0.003** | **0.003** | **0.003** | **0.010** | **0.005** | 0.098 | 0.337 | - | 0.209 |
| SRPC | 0.053 | 0.477 | 0.116 | 0.306 | 0.176 | **0.022** | **0.006** | 0.784 | 0.493 | 0.314 | 0.209 | - |
