# Supplementary material for: Diversity of compounds in femoral secretions of Galápagos iguanas (genera: Amblyrhynchus and Conolophus), and their potential role in sexual communication in lek-mating marine iguanas (Amblyrhynchus cristatus)
Source: PeerJ. 2017 Aug 17;5:e3689. doi: 10.7717/peerj.3689 (PMC5563446; doi:10.7717/peerj.3689)
Supplement: Supplemental Information 5 [file peerj-05-3689-s005.docx]

Table S4

Chemical distance (a) was calculated with the function vegdist of package “vegan”. Microsatellite data of 12 polymorphic nuclear loci was obtained from Macleod et al 2015 (available in Dryad data repository; http://dx.doi.org/10.5061/dryad. pp6bm). Genetic distance was calculated using function dist.genpop (package “adegenet”) on the microsatellite data. Both chemical distance and genetic distance were calculated for each population. Mantel correlation between chemical distance and genetic distance is showed in (b).

(a)

|  | ESP | FDA | FL | GEN | IS | SRL | MAR | PIN | SRPC | CRUZ | SFE | SAN |
| --- | --- | --- | --- | --- | --- | --- | --- | --- | --- | --- | --- | --- |
| ESP | 0.000 | 0.360 | 0.500 | 0.258 | 0.440 | 0.437 | 0.208 | 0.162 | 0.309 | 0.414 | 0.332 | 0.211 |
| FDA | 0.360 | 0.000 | 0.158 | 0.190 | 0.121 | 0.083 | 0.169 | 0.309 | 0.068 | 0.090 | 0.105 | 0.196 |
| FL | 0.500 | 0.158 | 0.000 | 0.309 | 0.079 | 0.095 | 0.296 | 0.423 | 0.198 | 0.098 | 0.201 | 0.325 |
| GEN | 0.258 | 0.190 | 0.309 | 0.000 | 0.275 | 0.254 | 0.124 | 0.158 | 0.171 | 0.255 | 0.140 | 0.193 |
| IS | 0.440 | 0.121 | 0.079 | 0.275 | 0.000 | 0.079 | 0.270 | 0.396 | 0.162 | 0.052 | 0.147 | 0.308 |
| SRL | 0.437 | 0.083 | 0.095 | 0.254 | 0.079 | 0.000 | 0.234 | 0.358 | 0.137 | 0.063 | 0.154 | 0.266 |
| MAR | 0.208 | 0.169 | 0.296 | 0.124 | 0.270 | 0.234 | 0.000 | 0.139 | 0.128 | 0.240 | 0.165 | 0.098 |
| PIN | 0.162 | 0.309 | 0.423 | 0.158 | 0.396 | 0.358 | 0.139 | 0.000 | 0.263 | 0.373 | 0.270 | 0.205 |
| SRPC | 0.309 | 0.068 | 0.198 | 0.171 | 0.162 | 0.137 | 0.128 | 0.263 | 0.000 | 0.124 | 0.099 | 0.175 |
| CRUZ | 0.414 | 0.090 | 0.098 | 0.255 | 0.052 | 0.063 | 0.240 | 0.373 | 0.124 | 0.000 | 0.143 | 0.282 |
| SFE | 0.332 | 0.105 | 0.201 | 0.140 | 0.147 | 0.154 | 0.165 | 0.270 | 0.099 | 0.143 | 0.000 | 0.208 |
| SAN | 0.211 | 0.196 | 0.325 | 0.193 | 0.308 | 0.266 | 0.098 | 0.205 | 0.175 | 0.282 | 0.208 | 0.000 |

(b)

Mantel statistic r: 0.05426

| **Mantel test** | **Permutations** | **R** | **P value** |
| --- | --- | --- | --- |
|  | 999 | 0.054 | 0.375 |
