# Supplementary material for: Diversity of compounds in femoral secretions of Galápagos iguanas (genera: Amblyrhynchus and Conolophus), and their potential role in sexual communication in lek-mating marine iguanas (Amblyrhynchus cristatus)
Source: PeerJ. 2017 Aug 17;5:e3689. doi: 10.7717/peerj.3689 (PMC5563446; doi:10.7717/peerj.3689)
Supplement: Supplemental Information 6 [file peerj-05-3689-s006.docx]

**Table S5**

Descriptive statistics showing male behavioural data and chemical profile. Average head bobbing rate (HB) was calculated per minute (see METHODS AND MATERIALS for details). The “Females” column represents the number of times a male was accompanied by females divided by the total number of observations. Chemical composition (%) shows the most influential lipophilic components: HA = Hexadecanoic acid; TA = Tetracosanoic acid; 9OA = 9-Octadecenoic acid, 11EA = 11-Eicosenoic acid. ID: Male identity.

|  | Behavioral variables | |  | Chemical composition (%) | | | |
| --- | --- | --- | --- | --- | --- | --- | --- |
| ID | HB rate | Females |  | HA | TA | 11EA | 9OA |
| 1 | 1.24 | 0/7 |  | 19.95 | 0 | 0 | 14.45 |
| 3 | 2.34 | 9/9 |  | 13.06 | 0.77 | 1.09 | 19.95 |
| 10 | 2.07 | 5/6 |  | 11.69 | 2.47 | 0.51 | 12.23 |
| 12 | 1.6 | 2/4 |  | 13.03 | 1.16 | 1.17 | 23.70 |
| 13 | 0.73 | 0/5 |  | 13.43 | 2.05 | 0 | 13.89 |
| 392 | NA | 1/6 |  | 14.44 | 1.93 | 0 | 15.47 |
| 393 | 1.8 | 7/7 |  | 10.45 | 2.57 | 1.15 | 13.07 |
| 396 | NA | 5/5 |  | 16.57 | 2.41 | 0.86 | 16.74 |
| 397 | 2.4 | 4/6 |  | 11.19 | 2.48 | 1.09 | 13.22 |
